# Supplementary figures and images for: Nr2f1a maintains atrial nkx2.5 expression to repress pacemaker identity within venous atrial cardiomyocytes of zebrafish
Source: eLife. 2023 May 15;12:e77408. doi: 10.7554/eLife.77408 (PMC10185342; doi:10.7554/eLife.77408)

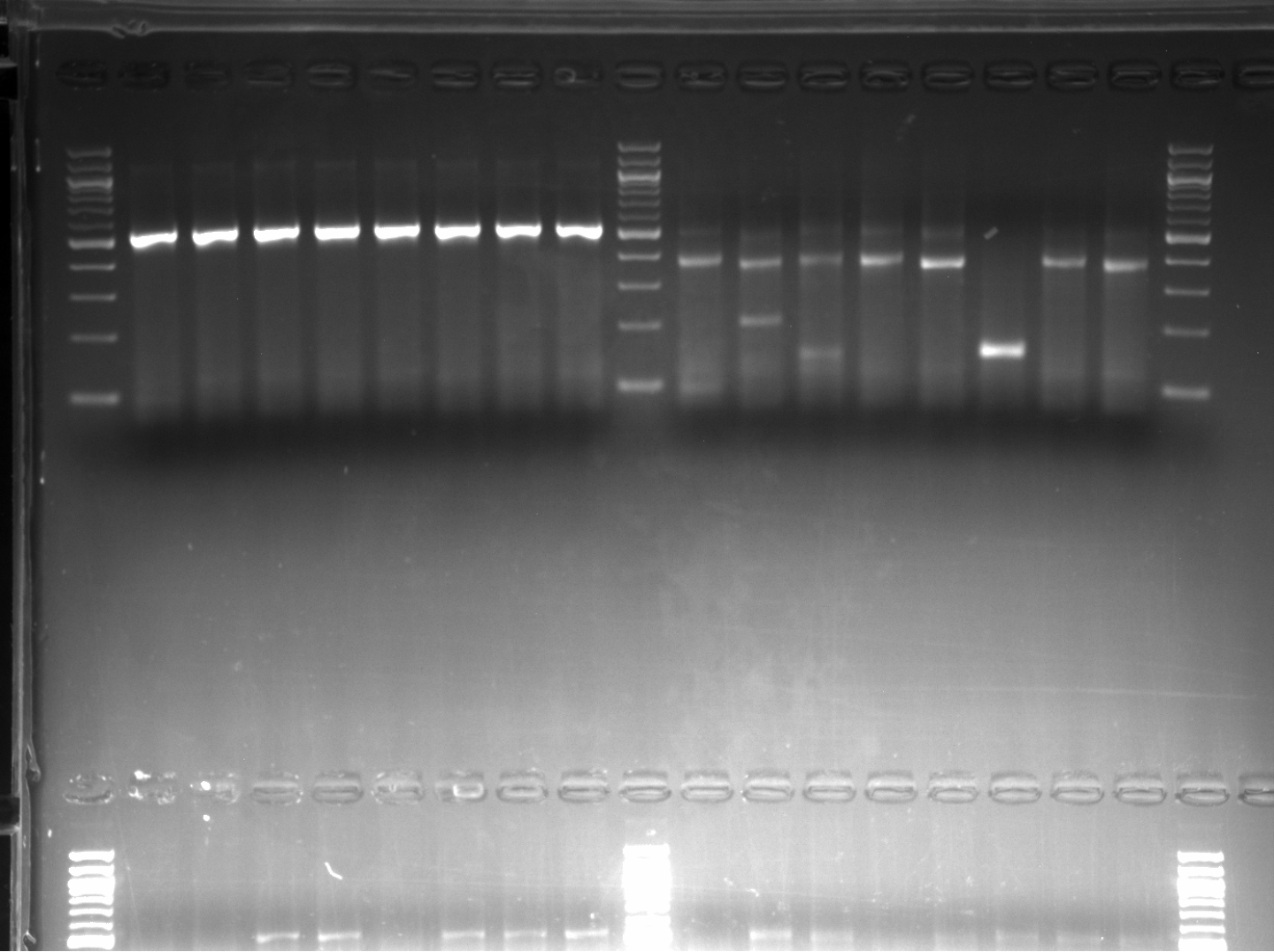

Supplement: Figure 6—figure supplement 2—source data 1. — Numbers in the labeled gels indicate individual control uninjected and CRISPR/Cas9-injected embryos. Control uninjected embryos 6–8 and CRISPR/Cas9-injected embryos 1–3 are shown in Figure 6—figure supplement 2. [file elife-77408-fig6-figsupp2-data1.zip › Figure 6 - figure supplement 2 - source data 1 - gel 1 .tif]

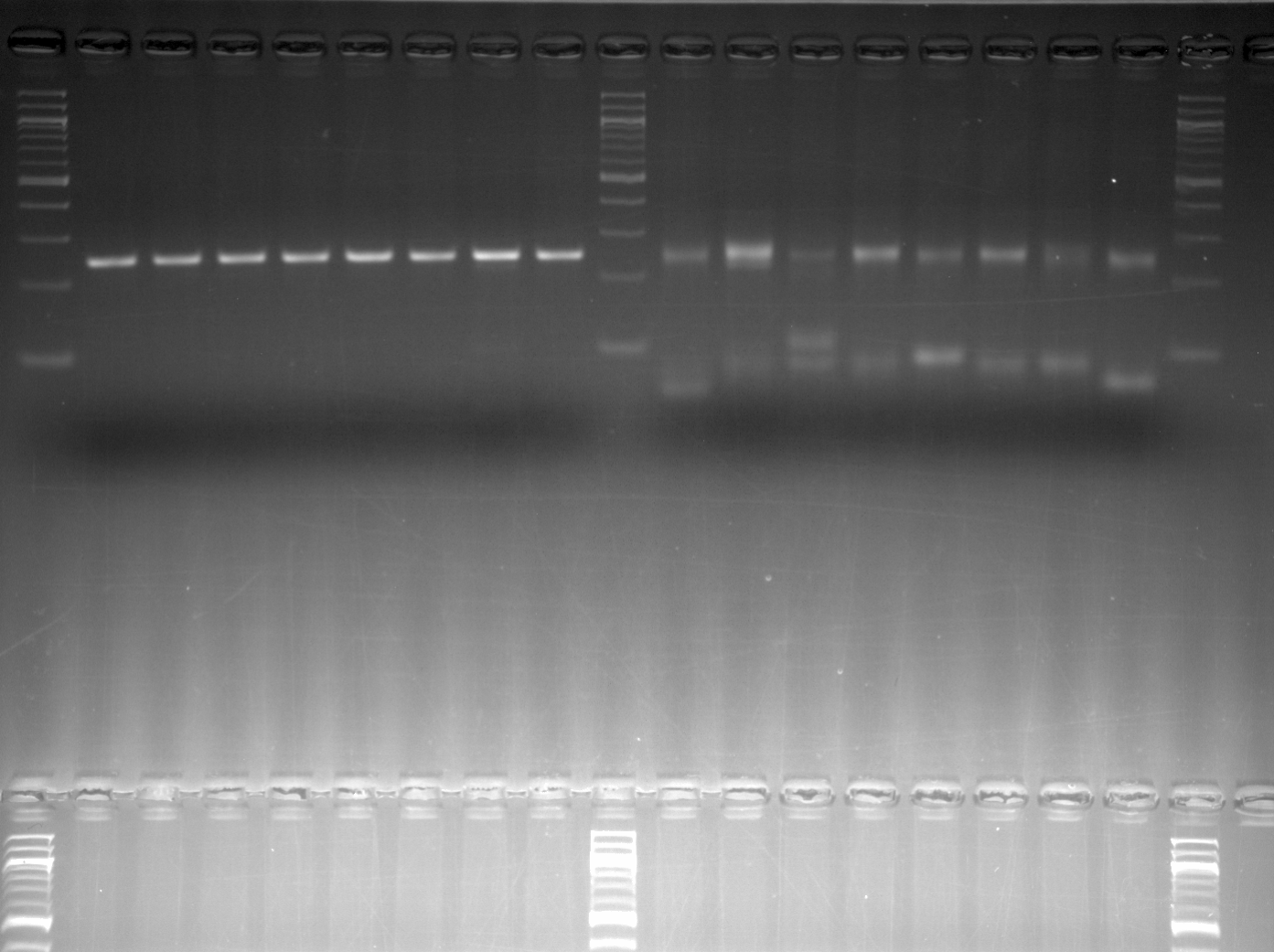

Supplement: Figure 6—figure supplement 2—source data 1. — Numbers in the labeled gels indicate individual control uninjected and CRISPR/Cas9-injected embryos. Control uninjected embryos 6–8 and CRISPR/Cas9-injected embryos 1–3 are shown in Figure 6—figure supplement 2. [file elife-77408-fig6-figsupp2-data1.zip › Figure 6 - figure supplement 2 - source data 1 - gel 2.tif]

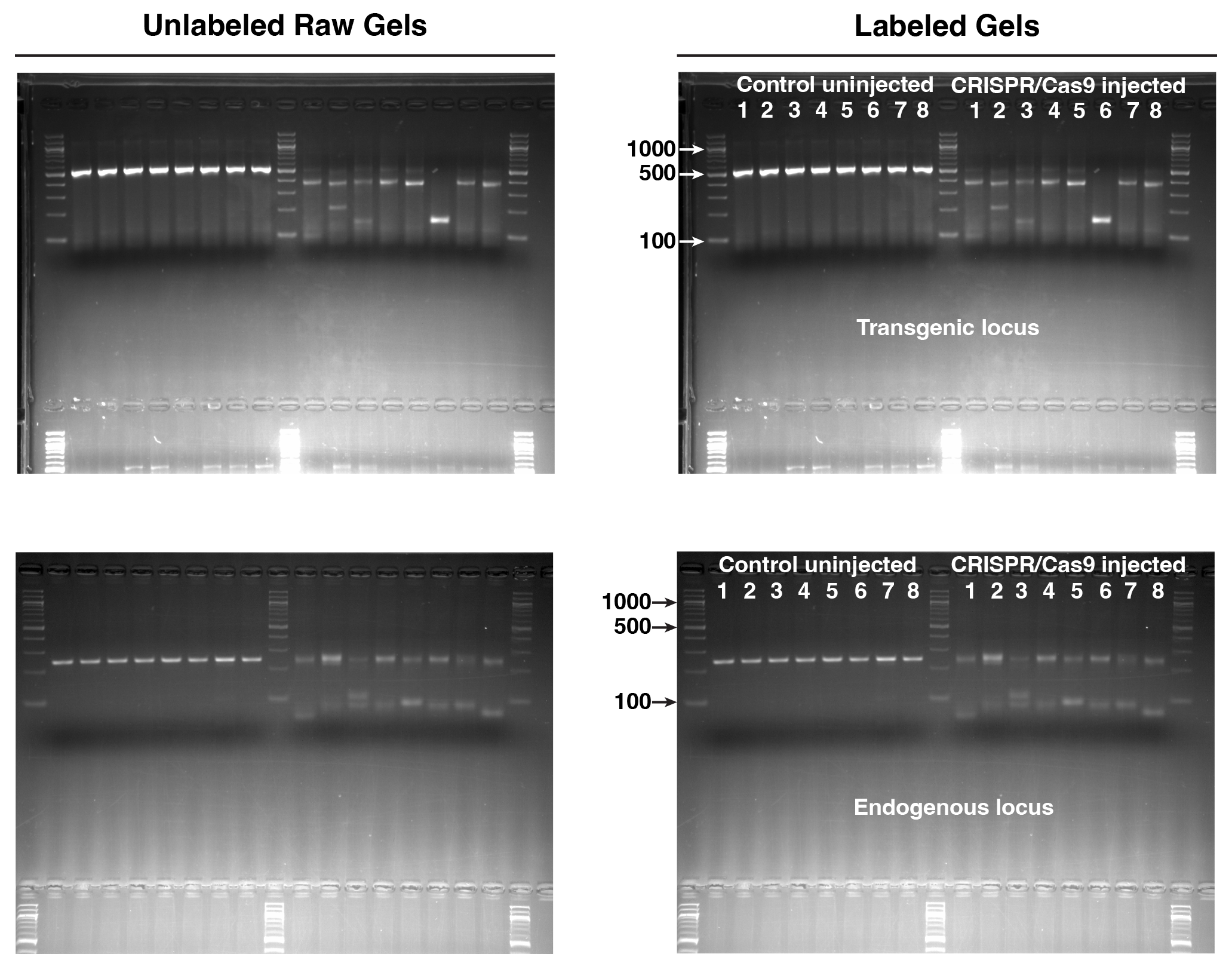

Supplement: Figure 6—figure supplement 2—source data 1. — Numbers in the labeled gels indicate individual control uninjected and CRISPR/Cas9-injected embryos. Control uninjected embryos 6–8 and CRISPR/Cas9-injected embryos 1–3 are shown in Figure 6—figure supplement 2. [file elife-77408-fig6-figsupp2-data1.zip › Figure 6 - figure supplement 2 - source data 1.tif]
